# Ethical issues in the use of genetic predictions of aggressive behavior in the criminal justice system: a systematic review

**DOI:** 10.3389/fgene.2025.1599750

**Published:** 2025-05-13

**Authors:** Pietro Refolo, Stefano Ferracuti, Simone Grassi, Costanza Raimondi, Giulia Mercuri, Massimo Zedda, Giovanni Aulino, Antonio Gioacchino Spagnolo, Antonio Oliva

**Affiliations:** ^1^ Department of Health Care Surveillance and Bioethics, Section of Bioethics and Medical Humanities, Università Cattolica del Sacro Cuore, Rome, Italy; ^2^ Research Centre for Clinical Bioethics and Medical Humanities, Università Cattolica del Sacro Cuore, Rome, Italy; ^3^ Department of Human Neurosciences, “Sapienza” University of Rome, Rome, Italy; ^4^ Department of Health Science, Section of Forensic Medical Sciences, University of Florence, Florence, Italy; ^5^ Department of Health Care Surveillance and Bioethics, Section of Legal Medicine, Università Cattolica del Sacro Cuore, Rome, Italy; ^6^ Fondazione Policlinico Universitario A. Gemelli IRCCS, Rome, Italy

**Keywords:** genetic testing, behavioral genetics, ethics, criminal justice, MAOA gene

## Abstract

**Background:**

The use of genetic predictions of aggressive behavior in the criminal justice system remains a subject of ongoing debate. Since behavioral genetic evidence is often used in criminal defense arguments, it is crucial to critically examine the ethical challenges associated with its application.

**Objective:**

This article seeks to identify and analyze these ethical concerns to ensure the responsible and equitable integration of genetic testing, when deemed necessary, into the judiciary system.

**Methods:**

A systematic review was conducted using PubMed, Web of Science, and Scopus, supplemented by manual searches of reference lists to identify additional relevant studies.

**Results:**

The search yielded 1,023 publications, 12 of which met the inclusion criteria. Seven key ethical concerns were identified: the risks of discrimination, stigmatization, eugenic reasoning, deterministic interpretations, overestimation of dangerousness, privacy violations, and medicalization, along with the risks posed by limited scientific literacy among legal professionals.

**Conclusion:**

The ethical challenges associated with genetic predictions of aggressive behavior underscore the need for a critical and multidisciplinary approach to their use in the criminal justice system. Collaboration among bioethicists, legal scholars, scientists, and communication experts is crucial to prevent misuse and reduce potential biases. Such an approach will help ensure that genetic insights are ethically applied, accurately interpreted, and used to promote justice rather than exacerbate systemic inequalities.

## 1 Introduction

Human behavioral genetics explores the origins of individual differences in psychological traits, such as intelligence and personality (Joseph, 2014). Its primary objective is to investigate the genetic foundations of behavior while accounting for the complex interaction between hereditary factors and environmental conditions (Goldsmith and Bihun, 1997).

The link between behavior and genetics, or heredity, can be traced back to the work of English scientist Sir Francis Galton (1822–1911), who introduced the concept of “nature and nurture” to describe the interplay between genetic inheritance and environmental influences.

A key focus of behavioral genetics is the investigation of genetic and environmental influences on violent behavior (Baker et al., 2006; Slutske, 2001; Viding, 2004). In the 1960s and 1970s, researchers hypothesized that the presence of an extra Y chromosome – known as XYY syndrome – heightened the risk of violent behavior. However, this theory was later discredited for its reliance on biased assumptions and flawed methodologies (Ashby, 1975; Steinfels and Levine, 1980).

A more promising link between genetic susceptibility and violent behavior emerged in 1993 when some researchers (Brunner et al., 1993) studied a Dutch family in which several male members exhibited mild cognitive impairments and impulsive aggression. Genetic analysis revealed a mutation on the X chromosome that deactivated the monoamine oxidase A (*MAOA*) enzyme, essential for neurotransmitter regulation. This study provided findings suggested that disruptions in neurotransmitter metabolism might contribute to aggressive tendencies.

In 2002, a study (Caspi et al., 2002) expanded on previous *MAOA* research, suggesting that even partial reductions in enzyme activity – linked to mutations in the gene’s promoter region – could heighten the risk of violent and antisocial behavior, particularly in unfavorable environments. As part of a large longitudinal study in Dunedin, New Zealand, researchers analyzed genetic variations in 442 males from a cohort of 1,037 individuals, using data collected up to age 26.

Subsequent studies have attempted to replicate the Dunedin findings, with most confirming the association, though some have not (Gold and Appelbaum, 2014); however, meta-analyses (Kim-Cohen et al., 2006; Byrd and Manuck, 2014) support the existence of the effect.

The *MAOA* gene has been cited in legal proceedings since its discovery, particularly in cases involving aggressive behavior. Already in 2017, a study (McSwiggan et al., 2017) identified 11 criminal cases where expert evidence on the *MAOA* gene was presented – nine in the US and two in Italy.

Behavioral genetics evidence is introduced in two legal contexts: in determining criminal responsibility and during the sentencing process. In particular, such evidence may be used to argue for diminished culpability, suggesting that a genetic predisposition to impulsivity or aggression could impair an individual’s capacity – defined as the ability to exercise self-control and make free and willful decisions. Given that capacity is a fundamental factor in assessing criminal liability, courts may consider an internal, uncontrollable drive toward aggression as grounds for excluding or mitigating culpability in cases of aggressive behavior. More commonly, behavioral genetics evidence is introduced during sentencing, where it serves as a mitigating factor, potentially influencing the severity of the punishment imposed (Berryessa et al., 2013; Oliva et al., 2021). For instance, in the United States, behavioral genetics has frequently been presented in capital cases, where defendants facing the death penalty have sought to use genetic predispositions to violent behavior as grounds for leniency (O’Mahony and de Paor, 2017).

However, the role of genetic evidence in the criminal justice system remains highly controversial, with scholars and legal experts offering differing perspectives on its implications (Sabatello and Appelbaum, 2017). While some argue that genetic predispositions to aggressive behavior can provide valuable insights into criminal responsibility and sentencing, others warn of the risks of misuse and misinterpretation. Indeed, the scientific robustness of such evidence remains a matter of debate, with concerns raised about its validity, reliability, and predictive power (Oliva et al., 2021). Moreover, the application of genetic predictions of aggressive behavior raises significant ethical challenges, as it intersects with fundamental principles of justice, fairness, and individual rights.

The aim of this article is to identify and critically examine the ethical issues associated with the use of genetic predictions of aggressive behavior in the criminal justice system. As the field of behavioral genetics continues to evolve, this analysis may contribute to ensuring its responsible and equitable integration into legal practice. This work is part of a broader research initiative titled “Genetic Predisposition to Aggressive-Impulsive Antisocial Behavior: Forensic Aspects”, funded by the European Union (NextGenerationEU) and the Italian Ministry of University and Research.

To date, no comprehensive analysis has systematically explored the ethical issues related to the use of genetic predictions of aggressive behavior in the criminal justice system. To the best of our knowledge, this systematic review represents the first attempt to fully map the ethical debate surrounding the application of genetic predictions of aggressive behavior in legal proceedings.

## 2 Methods

This study aims to explore the ethical issues arising from the use of genetic predictions of aggressive behavior in the criminal justice system. To ensure methodological rigor and transparency, the study was designed and reported in accordance with the Preferred Reporting Items for Systematic Reviews and Meta-Analys (PRISMA)-Ethics Reporting Guidelines (Kahrass et al., 2021).

Prior to conducting the study, four members of the research team (PR, SF, SG and AO) developed a structured protocol outlining the research objectives, search strategy, and inclusion criteria. The protocol was subsequently reviewed and approved by all researchers during a dedicated meeting on 1 August 2024. Given the thorough internal validation process, formal protocol registration was deemed unnecessary. A comprehensive literature search was conducted across three major academic databases: PubMed, Web of Science, and Scopus. These databases were selected for their broad interdisciplinary coverage, ensuring a thorough examination of the existing literature.

The search strategy was organized into three main thematic areas: the first centered on terminology related to “genetic predictions”, the second on “aggressive behavior”, and the third on “ethical issues”. To enhance search accuracy and scope, synonyms and alternative spellings for key terms were incorporated into each category. The PubMed search strategy is detailed in Table 1 and was subsequently adapted for Web of Science and Scopus.

**TABLE 1:** Search string.

Database	Search string
PubMed	(((((((((“Sociobiology” [Mesh]) OR “Genetics” [Mesh]) OR “genetics” [Subheading]) OR “Forensic Genetics” [Mesh]) OR “Genetics, Behavioral” [Mesh]) OR “Human Genetics” [Mesh]) OR “Genes” [Mesh]) OR “Brunner Syndrome” [Supplementary Concept]) AND (((“Violence” [Mesh] AND “Domestic Violence” [Mesh]) OR “Aggression” [Mesh]) OR “Disruptive, Impulse Control, and Conduct Disorders” [Mesh]OR aggressive OR aggression OR violent OR violence)) AND ((“Ethics” [Mesh] OR “ethics” [Subheading] OR “Ethics, Clinical” [Mesh] OR “Bioethics” [Mesh]) OR “Morals” [Mesh] OR ethic*)

The search was limited to English-language publications. All database searches were conducted in November 2024. Additionally, a manual review was performed, including an examination of reference lists from the selected studies to identify any additional relevant literature.

The inclusion and exclusion criteria were established before conducting the search (Table 2). Studies were considered eligible if they explicitly focused on behavioral genetics, included a clear reference to aggressive behavior, and directly engaged with ethical considerations concerning the use of genetic predictions of aggressive behavior in the criminal justice system. Studies were excluded if they lacked a forensic context, did not directly discuss aggressive behavior, or failed to provide a clear ethical reference on genetic predictions in legal settings.

**TABLE 2:** Inclusion and exclusion criteria.

Inclusion criteria	Exclusion criteria
- Focus on behavioral genetics - Explicit reference to aggressive behavior - Direct engagement with ethical considerations regarding the use of genetic predictions of aggression in legal settings	- The study addressed behavioral genetics but did not make any reference to the forensic context - The study lacked a direct discussion on aggressive behavior - The study did not address ethical considerations related to the implications of genetic predictions in legal settings

Two independent reviewers (PR and CR) conducted the study screening process using Rayyan software^1^. The software facilitated the identification and removal of duplicate records, with each duplicate manually verified by both reviewers. Titles and abstracts of the retrieved documents that met the inclusion criteria were assessed separately by the reviewers. Disagreements were resolved through discussion, and unresolved cases were escalated to a third reviewer (AGS) for adjudication.

For the full-text review, PR and CR independently analyzed each study at least twice before extracting data to ensure a thorough understanding of the content. The articles were evaluated to identify ethical arguments related to the use of genetic predictions of aggressive behavior in the criminal justice system. A third researcher (AGS) cross-checked the extracted data for accuracy. Any inconsistencies detected during this process were resolved through consultation of the primary study documents and discussions within the research team. The extracted data are detailed in the Supplementary Material S1. Given the lack of a standardized framework for evaluating ethical reasoning, no formal quality appraisal of the selected studies was conducted.

The synthesis process followed a critical interpretive synthesis approach (Dixon-Woods et al., 2006). This process enabled the research team to identify a coherent set of recurring concerns, which were further examined and refined through a series of iterative discussions. Each identified theme was subsequently developed into a dedicated paragraph, representing a distinct ethical domain. These thematic categories capture the central ethical tensions emerging from the literature and provide the analytical framework through which the results are organized and interpreted.

## 3 Results

**FIGURE 1:**
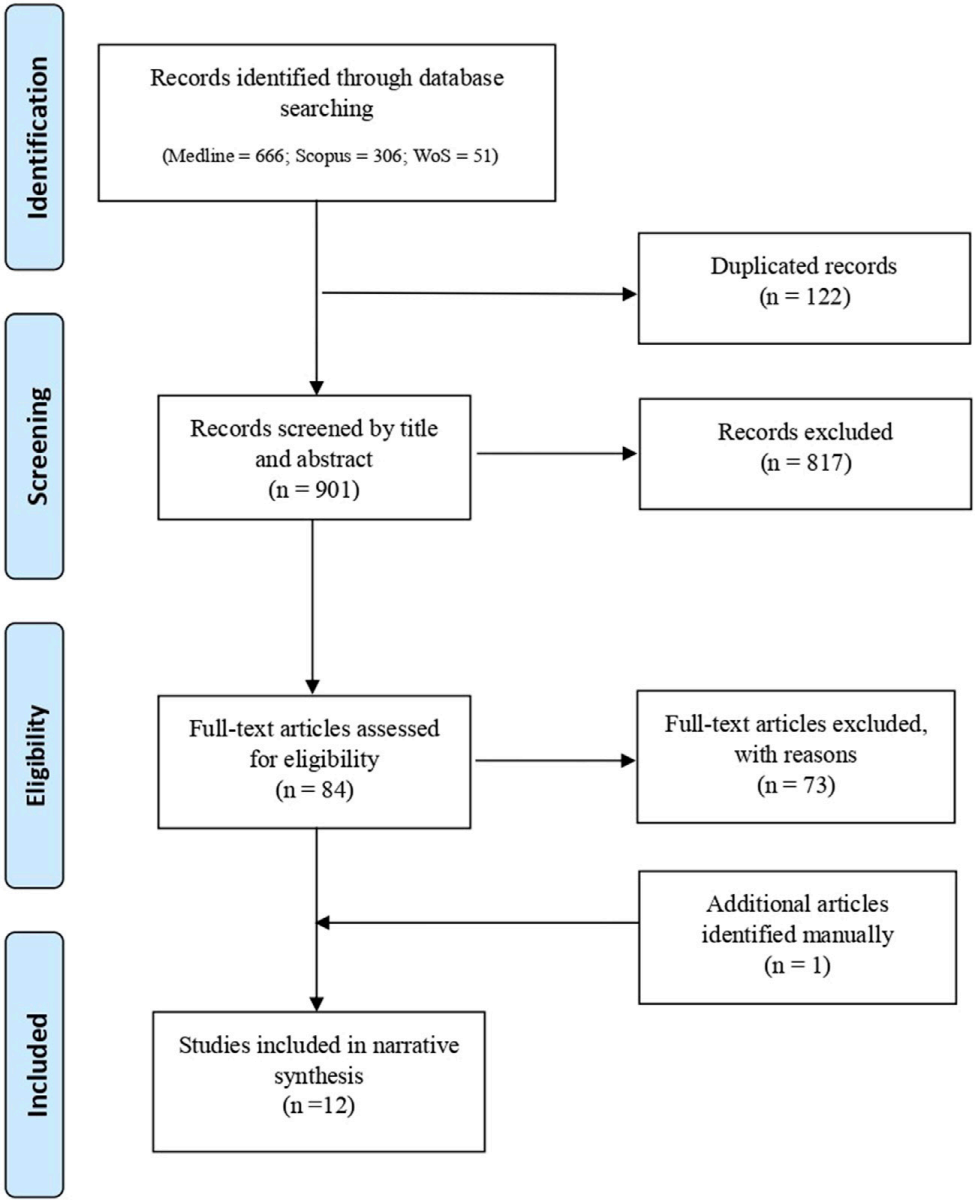
PRISMA flow diagram.

The search process initially identified 1,023 records, and 122 duplicates were subsequently removed. During the preliminary screening phase, 817 records were excluded based on their titles and/or abstracts, as they did not meet the eligibility criteria. Although 84 full-text articles were assessed in the second-level screening, only 11 were ultimately included in the final analysis. The primary reasons for exclusion at this stage included ethical discussions that remained overly general (n = 31); studies that, although they referred to the legal domain, addressed ethical concerns in areas unrelated to criminal justice – such as education, employment, or healthcare (n = 22); and contributions that relied on outdated conceptual or ethical frameworks, limiting their relevance to current debates (n = 20). Additionally, one more article was identified through reference list screening, bringing the final selection to 12 studies (Wasserman, 2004; DeCamp and Sugarman, 2004; Rothstein, 2005; Popma and Raine, 2006; Savulescu and et al., 2006; Levitt and Manson, 2007; Berryessa et al., 2013; O’Mahony and de Paor, 2017; Specker et al., 2017; Ferioli and Picozzi, 2018; Glenn and McCauley, 2019; Meurer, 2021). A PRISMA flow diagram represented in Figure 1 illustrates the study selection process.

The recurring concerns identified in the interpretative analysis coalesced into the following thematic categories: discrimination, stigmatization, resurgence of eugenic thinking, genetic determinism, overestimation of dangerousness, infringements on privacy, medicalization, and risks linked to limited scientific expertise. Below, each of these categories is discussed in detail.

### 3.1 Discrimination

Discrimination is a widely recognized ethical concern in discussions on the use of genetic predictions of aggressive behavior particularly within the criminal justice system (Wasserman, 2004; Rothstein, 2005; Popma and Raine, 2006; Savulescu and et al., 2006; Levitt and Manson, 2007; Berryessa et al., 2013; O’Mahony and de Paor, 2017; Specker et al., 2017; Ferioli and Picozzi, 2018; Glenn and McCauley, 2019). The mere identification of a genetic predisposition to violent behavior may result in social and institutional exclusion, reinforcing biases and limiting opportunities.

In the workplace, the misuse of genetic information may result in individuals being unjustly denied employment or career advancement based on perceived genetic risks rather than actual skills and competencies. This undermines the principles of merit-based hiring and professional development, further entrenching systemic inequalities. The fear of liability or workplace disruptions may drive employers to make preemptive decisions that disadvantage individuals with certain genetic profiles, even in the absence of any actual performance concern.

Similarly, in the insurance sector, concerns arise over whether private insurers should be allowed to incorporate genetic predispositions into risk assessment. Such practices could lead to higher premiums or outright denial of coverage for individuals who have never exhibited aggressive behavior, effectively penalizing them for factors beyond their control. More broadly, in healthcare, the integration of behavioral genetics into medical decision-making raises ethical concerns about the potential shift from a patient-centered approach to a predictive model that prioritizes risk management over individualized treatment.

Finally, discrimination could also extend to education, where students labeled as “at risk” based on their genetic profiles may face reduced opportunities. If schools begin to use genetic predispositions as indicators of academic potential or behavioral tendencies, they may limit access to advanced programs, alter teaching methods, or impose additional monitoring on certain students, regardless of their actual performance or behavior. This could undermine the principle of equal educational access and deepen existing disparities, disproportionately affecting marginalized communities. By placing undue weight on genetic predispositions, educational institutions risk overlooking the significant role of personal effort, social context, and educational support in shaping student outcomes.

### 3.2 Stigmatization

A second ethical concern regarding the use of genetic predictions of aggressive behavior in the criminal justice system is the risk of stigmatization (DeCamp and Sugarman, 2004; Rothstein, 2005; Levitt and Manson, 2007; Berryessa et al., 2013; O’Mahony and de Paor, 2017; Specker et al., 2017; Ferioli and Picozzi, 2018; Glenn and McCauley, 2019; Meurer, 2021). Classifying defendants as “at risk” based on genetic markers has the potential to reinforce existing biases, shaping not only legal outcomes but also broader societal perceptions of criminality. Such labeling may contribute to disparities in sentencing, access to rehabilitative programs, and parole decisions, perpetuating cycles of exclusion rather than fostering justice.

Beyond legal consequences, genetic labeling can have profound psychological effects on those subjected to it. Being identified as having a genetic predisposition to antisocial behavior may undermine an individual’s self-esteem, personal identity, and sense of agency, fostering internalized stigma and social alienation. Those labeled as genetically “high risk” may struggle with feelings of inevitability or hopelessness, believing their genetic makeup dictates their future. This psychological burden can further obstruct reintegration efforts, as individuals with such labels may face social isolation both within correctional institutions and in society at large.

A particularly concerning aspect is the reinforcement of negative stereotypes, especially against already disadvantaged and marginalized communities. If genetic or epigenetic markers associated with behavioral traits are more frequently identified within specific socioeconomic or ethnic groups, their use in risk assessment may inadvertently legitimize harmful prejudices. This could exacerbate social and health inequalities by justifying disproportionate surveillance, policing, or punitive measures against certain populations under the guise of crime prevention. Rather than advancing justice, such applications of genetic screening risk becoming instruments of social control, reinforcing existing disparities and deepening mistrust in the legal system.

To mitigate these risks, ethical frameworks must prioritize the no-stigma principle, ensuring that genetic insights do not translate into social exclusion or psychological harm.

### 3.3 Resurgence of eugenic thinking

The increasing reliance on genetic information to isolate and target specific behavioral traits raises serious concerns about the revival of eugenic-style policies and attitudes (Wasserman, 2004; Savulescu et al., 2006; O’Mahony and de Paor, 2017). Historically, eugenic ideologies sought to classify individuals based on perceived biological “fitness,” often leading to coercive interventions, forced sterilizations, and systematic discrimination against marginalized groups. While contemporary genetic research aims to enhance the understanding of human behavior, its application in predictive justice risks reintroducing similar patterns of exclusion and control under a scientific guise.

To mitigate these ethical risks, strict regulatory and ethical safeguards must be implemented to prevent the misuse of genetic data in ways that echo eugenic ideologies. Furthermore, the ethical application of genetic insights in the criminal justice system should be subject to interdisciplinary oversight, meaning that decisions must be evaluated and guided by a diverse group of experts–including bioethicists, legal scholars, sociologists, and advocates for social justice. This collaborative supervision ensures that the development and use of genetic tools are not driven solely by scientific or technological considerations, but are also informed by ethical, legal, and societal perspectives. Such a pluralistic approach is essential to avoid repeating past injustices and to prevent the emergence of new forms of social control disguised as scientific progress.

### 3.4 Determinism

A fourth ethical issue concerns the implications of genetic predictions for free will and legal accountability (Wasserman, 2004; Popma and Raine, 2006; Savulescu et al., 2006; Levitt and Manson, 2007; Berryessa et al., 2013; O’Mahony and de Paor, 2017; Specker and et., 2017; Ferioli and Picozzi, 2018). The integration of biological factors–such as genetic markers associated with impulse control–challenges traditional conceptions of moral and legal responsibility. If certain individuals exhibit biological deficits that impair their capacity for self-regulation, should their culpability for criminal actions be reconsidered?

One of the primary risks is the reinforcement of deterministic assumptions about antisocial behavior. Perceiving criminal tendencies as innate or inherited could contribute to a self-fulfilling prophecy in which individuals labeled as genetically “predisposed to aggressive behavior” are treated as future offenders regardless of their actual conduct. Such labeling may influence legal decisions, resulting in harsher sentencing, prolonged surveillance, or restricted access to rehabilitation programs based on perceived rather than actual risk. Moreover, this perspective risks diverting attention from the social and environmental factors that contribute to criminal behavior, reinforcing a reductionist view of crime as biologically predetermined rather than as the outcome of complex social, psychological, and economic influences.

A particularly concerning development is the potential shift toward a preventive model of justice. If specific genetic or neurological markers are considered adequate grounds for state intervention, the legal system may start imposing restrictions on individuals based on predictive assessments rather than proven offenses. This could lead to increased monitoring, mandatory treatment, or even preventive detention for individuals deemed “at risk” of criminal behavior, despite the absence of any unlawful conduct. This approach mirrors historical frameworks rooted in biological determinism, such as the 19th-century theories of criminal anthropology that attempted to classify “born criminals” based on physiological traits–a concept long discredited but now resurfacing in a more scientifically refined guise.

The ethical and legal consequences of such a shift would be profound. A system that legitimizes restrictions on individual liberty based on probabilistic genetic assessments risks violating core principles of justice, including the presumption of innocence and the right to due process.

### 3.5 Overestimation of the dangerousness

The integration of genetic screening into the criminal justice system raises significant concerns about the potential overestimation of the predictive value of genetic markers for violent behavior and, consequently, the perceived dangerousness of individuals (Rothstein, 2005; Berryessa et al., 2013; O’Mahony and de Paor, 2017). Courts and policymakers may be misled into assuming a stronger causal relationship between genetic traits and criminal tendencies than what is supported by scientific evidence.

A particularly troubling aspect is the potential labeling of individuals as future criminals based on genetic screening. If genetic predispositions are treated as deterministic indicators of future conduct, individuals could be subjected to heightened surveillance, restricted opportunities, or even preemptive legal measures despite the absence of any criminal behavior. This approach not only undermines the presumption of innocence but also penalizes individuals, limiting their social mobility and reinforcing systemic biases.

### 3.6 Privacy infringement

The collection and storage of genetic data in DNA databases raise serious ethical concerns, particularly regarding consent, confidentiality, and the potential misuse of sensitive information (Wasserman, 2004; Rothstein, 2005; Berryessa et al., 2013; O’Mahony and de Paor, 2017; Ferioli and Picozzi, 2018; Glenn and McCauley, 2019). Given the uniquely personal and immutable nature of genetic data, privacy protections must be rigorous to prevent unauthorized access, disclosure, and exploitation. Without robust safeguards, genetic information could be misused in ways that extend far beyond the original forensic purposes, posing critical challenges to the balance between security, individual rights, and ethical governance.

Moreover, the potential for misuse and secondary use of genetic data heightens concerns about discrimination and unjustified surveillance. Unauthorized access to genetic databases – whether by state authorities, private entities, or malicious actors – could lead to the exploitation of genetic profiles for purposes beyond criminal justice, including employment decisions, insurance coverage, or predictive assessments of behavior. Such applications risk reinforcing systemic biases, disproportionately affecting marginalized communities and exacerbating existing social inequalities.

The psychosocial impact of privacy breaches also warrants serious consideration. The unauthorized disclosure of genetic information can have profound consequences for individuals and their families, leading to stigma, emotional distress, and social marginalization. The awareness that one’s genetic data is permanently stored and potentially accessible by various institutions may contribute to a climate of fear and distrust in both the legal system and broader societal structures.

To address these ethical concerns, strict privacy protections and regulatory oversight must be implemented. This includes enforcing explicit and informed consent procedures, implementing data encryption and access controls, and establishing clear guidelines on the retention and deletion of genetic records. Additionally, policies must be developed to limit the scope of DNA database usage, ensuring that genetic data is employed strictly within legal and ethical boundaries and preventing its expansion into areas that could infringe upon fundamental rights.

Incorporating these safeguards is essential to maintaining trust in the forensic applications of genetics while upholding the principles of justice, autonomy, and privacy. Without comprehensive protections, the widespread use of DNA databases risks evolving into a tool for excessive state surveillance and social control, ultimately undermining the ethical foundations of the criminal justice system.

### 3.7 Medicalization

The increasing reliance on genetic and neurobiological insights in criminal justice raises concerns about the medicalization of antisocial behavior, that is the process of redefining behaviors traditionally viewed as moral or social transgressions as medical conditions requiring clinical intervention (DeCamp and Sugarman, 2004; Berryessa et al., 2013; Specker et al., 2017).

One of the primary concerns is that medicalization could undermine personal responsibility. If antisocial or violent tendencies are classified as medical disorders, defendants may increasingly be seen as patients, raising questions about the extent to which they should be held legally accountable for their actions.

Another critical issue is the use of pharmacological or neurological interventions to manage norm-defiant behavior. Treating individuals labeled as predisposed to criminal behavior with medication – whether to suppress aggression, enhance impulse control, or modify other traits – raises ethical concerns about autonomy and informed consent. The potential for coercive treatment is particularly troubling, as individuals within the criminal justice system may face pressure to undergo medical interventions as a condition for parole, reduced sentencing, or rehabilitation. This echoes past controversies surrounding forced medication, chemical castration, and other state-imposed biomedical interventions, which were often justified as measures to protect public safety but frequently resulted in serious human rights violations.

Furthermore, the expansion of medical interventions in criminal justice risks creating a system in which behavioral control takes precedence over addressing the underlying social and structural causes of crime. By attributing antisocial behavior primarily to biological or neurological dysfunctions, medicalization may divert attention from critical social, economic, and psychological factors that contribute to criminality. This could lead to an over-reliance on biomedical solutions, while neglecting the broader need for legal, educational, and social reforms that aim to prevent crime and rehabilitate offenders.

### 3.8 Risks of limited scientific expertise

The increasing use of genetic evidence in criminal justice raises serious concerns about the scientific literacy of judges, juries, and legal professionals. While genetic insights can provide valuable information in forensic investigations, the complexity of genetics presents significant challenges for those responsible for interpreting its findings. A lack of expertise among legal decision-makers increases the risk of misinterpretation, over-reliance on expert testimony, and flawed judicial outcomes, potentially leading to miscarriages of justice (Rothstein, 2005; Berryessa et al.).

One of the primary concerns is that judges and juries often lack the necessary scientific background to critically evaluate genetic evidence. Unlike traditional forensic evidence, genetic data is highly technical and probabilistic in nature. Courts may struggle to assess the validity, reliability, and limitations of genetic findings, particularly in behavioral genetics, which remains an evolving and controversial field. This gap in understanding creates a dangerous reliance on expert testimony, where the persuasive power of scientific authority may overshadow a nuanced consideration of the evidence. Without adequate scientific literacy, legal decision-makers may either overestimate the certainty of genetic predictions, leading to unjustified conclusions about an individual’s criminal responsibility, or dismiss legitimate findings due to skepticism or misunderstanding.

Another key concern is the risk of biased or misleading expert testimony. While expert witnesses play a crucial role in translating complex genetic information for the court, their interpretations may vary based on differing scientific perspectives, institutional affiliations, or even unconscious biases. In adversarial legal systems, experts may present conflicting interpretations, leaving judges and juries to choose between competing narratives without the necessary knowledge to critically assess their scientific validity. This increases the risk that legal decisions may be driven more by rhetoric than by sound scientific reasoning, ultimately undermining the fairness of the judicial process.

The absence of standardized guidelines for the use of genetic evidence further exacerbates these challenges. Unlike traditional forensic disciplines with well-established methodologies, behavioral genetics and predictive genetic profiling continue to be areas of active debate. The lack of clear legal and ethical frameworks governing the admissibility and interpretation of genetic findings creates inconsistencies in how such evidence is applied across different cases and jurisdictions, increasing the likelihood of arbitrary or unjust outcomes.

## 4 Discussion

The *MAOA* gene has been referenced in judicial proceedings since its identification, especially in cases related to aggressive behavior.

One of the earliest and most notable cases was *Mobley v. State* (1995), marking the first time the *MAOA* gene was discussed in a U.S. courtroom. A significant milestone in Europe occurred in *Bayout v. Francesco* (2009), where an Italian court reduced a convict’s sentence based on genetic predisposition to violent behavior, making it the first instance in which behavioral genetics influenced a legal ruling on the continent. Additionally, *State v. Waldroup* (2011) in the United States further underscored the role of genetic evidence in criminal trials (O’Mahony and de Paor, 2017).

In addition to the *MAOA* gene, several other genes have been associated with antisocial and aggressive behavior. These include *DAT-1* (Dopamine Transporter 1), which regulates dopamine transmission; *BDNF* (Brain-Derived Neurotrophic Factor), which is crucial for brain function; and *CRHBP* (Corticotropin-Releasing Hormone Binding Protein), which influences stress response. More recent studies (Assari et al., 2018; Musci et al., 2019; Koyama et al., 2024) have also linked the low-activity variants of *5HTTLPR* (Serotonin Transporter Linked Polymorphic Region, part of the *SLC6A4* gene regulating serotonin levels), the 7-repeat allele of *DRD4* (Dopamine Receptor D4, associated with impulsivity), the A1 allele of *DRD2* (Dopamine Receptor D2, involved in reward processing), the *H3* (*GGA*) haplotype of *CRHR1* (Corticotropin-Releasing Hormone Receptor 1, influencing stress response), and specific variants of *COMT* (Catechol-O-Methyltransferase), particularly the *Val158Met* polymorphism, which affects dopamine metabolism, with violent tendencies.

However, the debate over the use of genetic predictions of behavior remains controversial, particularly in discussions surrounding criminal responsibility. It is no coincidence that in the early stages of behavioral genetics research, scientific conferences addressing the topic were met with protests and opposition, reflecting widespread concerns about the potential misuse of genetic explanations for social and legal purposes (Birch, 1995).

Historically, fears of biological determinism have been linked to discriminatory policies, racial bias, and the erosion of personal responsibility, leading many scholars and activists to challenge the legitimacy of genetic predictions in forensic contexts. This controversy remains relevant today, as the application of behavioral genetics in criminal justice risks reviving outdated notions of “born criminals”, thereby reinforcing biases rather than fostering a nuanced understanding of crime as a multidimensional phenomenon.

The review conducted in our study allowed us to identify several critical ethical concerns that were both present and emphasized in the selected articles. These concerns reflect and resonate with the main ethical frameworks that have been well established in the field of genetics. Among the most prominent issues are the risks of discrimination, stigmatization, eugenic thinking, deterministic interpretations, overestimation of dangerousness, privacy violations, medicalization, and the potential consequences of limited scientific expertise in legal decision-making.

Beyond these specific considerations, we argue that the core of this ethical debate centers on scientific reductionism – the tendency to oversimplify complex human behaviors by attributing them primarily to genetic or biological factors (Newson, 2004; Dick, 2011). While reductionist approaches once dominated scientific discourse, they are now widely recognized as inadequate for capturing the intricate interplay between genetics, environment, and individual agency. The findings of this study reinforce the argument that predicting violent behavior solely based on genetic markers is scientifically flawed and ethically problematic. The challenge, therefore, lies in rejecting simplistic genetic explanations and adopting a more holistic, interdisciplinary approach that accounts for psychosocial, cultural, and environmental influences on behavior.

A particularly critical element for contemporary societies is the impact on public opinion. One of the greatest risks associated with genetic predictions of aggressive behavior is the public misinterpretation of scientific findings. If genetic predispositions to criminal behavior are presented without appropriate context, they could fuel misconceptions, fear, and social stigma, leading to harmful policies that undermine human rights and justice. The sensationalization of genetic research in media narratives could contribute to moral panic, reinforcing stereotypes about certain populations and justifying coercive legal measures based on speculative risk assessments. Therefore, clear, transparent, and responsible communication is essential to ensuring that both the public and policymakers understand the limitations of behavioral genetics and do not misapply genetic data in ways that perpetuate discrimination and social control (Meurer, 2021; Ferioli and Picozzi, 2018).

Despite these risks, we do not argue for the exclusion of genetic data from legal processes. On the contrary, we believe that its responsible use is possible – provided it is embedded within a robust ethical framework and interpreted through a multidisciplinary lens. Genetic evidence should inform but never determine legal outcomes, and its use must be guided by principles that protect individual rights and promote justice.

Based on the findings of our review, we propose the following recommendations for legal practitioners, particularly in complex cases involving aggressive behavior and suspected genetic predispositions:

• Contextual interpretation: genetic predispositions must never be treated as deterministic. Legal reasoning must consider social, psychological, and environmental factors.
• Scientific training: judges and legal professionals should receive adequate training in behavioral genetics and related disciplines to evaluate scientific claims critically.
• Interdisciplinary oversight: courts should consult advisory panels composed of bioethicists, neuroscientists, legal experts, and psychologists to assess the appropriateness and ethical implications of using genetic data in specific cases.
• Non-discrimination: legal decisions must avoid stigmatizing individuals based on genetic profiles and ensure that such information is not used to justify unequal treatment.
• Standards for expert testimony: clear and consistent criteria are needed to govern the admissibility and reliability of genetic evidence in court.
• Privacy protection: strict safeguards must be applied to the collection, storage, and use of genetic information, including informed consent and limitations on data sharing.
• Transparent communication: Both the legal system and media should adopt clear and responsible strategies for communicating the meaning and limitations of genetic findings.
• Rejection of genetic-based preventive justice: measures taken against individuals must be grounded in conduct, not in probabilistic genetic assessments.

In conclusion, the ethical concerns surrounding genetic predictions of aggressive behavior underscore the importance of maintaining a critical perspective on how genetic findings are integrated into the legal system. Moving forward, a multidisciplinary approach – involving bioethicists, legal scholars, scientists, and communication experts – is crucial to ensuring that genetic insights are used ethically, interpreted accurately, and applied in ways that promote justice rather than reinforce systemic inequalities. Practically, this means establishing institutional frameworks for ongoing ethical review, such as advisory committees that include diverse disciplinary perspectives to evaluate the admissibility and use of genetic evidence in courtrooms. Legal professionals should be trained in the interpretation of scientific findings, while scientists should collaborate with ethicists to assess the societal impact of their work. Communication experts, particularly those specialized in science communication, can play a key role in translating complex genetic information for judges, juries, policymakers, and the general public, helping to prevent misinterpretations that could lead to moral panic or policy distortions.

This collaborative effort would not only ensure responsible integration of behavioral genetics into legal and policy frameworks, but also foster transparency, public trust, and the protection of fundamental human rights.

We acknowledge that this review has at least three significant limitations.

First, the way in which our research question was formulated may have inadvertently excluded some relevant studies that could have provided additional perspectives on the topic. The specific criteria used to define the scope of this review may have influenced the selection process, potentially limiting the diversity of viewpoints considered.

Second, our literature search was conducted using only three databases, which increases the possibility of having overlooked significant studies. This constraint may have led to the omission of research that could have enriched our analysis with alternative data or interpretations, thereby affecting the overall depth and comprehensiveness of our findings.

Third, most of the studies included in this review primarily reflect perspectives from the United States and Europe. This geographic concentration limits the applicability of our conclusions to a broader international context, as findings may not fully capture cultural, legal, or ethical nuances present in other regions.

To overcome these limitations, future research should expand the search methodology by incorporating a wider range of databases and adopting a more inclusive approach to study selection. Additionally, greater efforts should be made to integrate research from underrepresented regions to ensure a more comprehensive and globally relevant analysis.

## 5 Conclusion

Based on the findings of our review, the use of genetic predictions of aggressive behavior in the criminal justice system raises profound ethical concerns. These include the risks of discrimination, stigmatization, eugenic thinking, deterministic interpretations, overestimation of dangerousness, privacy violations, medicalization, and the potential consequences of limited scientific expertise in legal decision-making.

To navigate these challenges responsibly, a more holistic and interdisciplinary approach is essential. Rather than relying on reductionist explanations, future research and policy should integrate insights from genetics, neuroscience, psychology, sociology, and legal studies to develop a more nuanced understanding of crime and aggressive behavior. This approach must also be accompanied by robust ethical safeguards to prevent misuse and ensure that genetic research contributes to justice rather than reinforcing biases and inequalities.

Ultimately, these efforts are crucial to avoiding the mistakes of the past, where misguided applications of science have fueled injustice, discrimination, and ethical violations.

## Data Availability

The original contributions presented in the study are included in the article/Supplementary Material, further inquiries can be directed to the corresponding authors.

## Footnotes

1 Accessed on 10 January 2025 at: https://www.rayyan.ai/, accessed on 10 January 2025.
