# Supplementary material for: Ethical issues in the use of genetic predictions of aggressive behavior in the criminal justice system: a systematic review
Source: Front Genet. 2025 May 13;16:1599750. doi: 10.3389/fgene.2025.1599750 (PMC12106298; doi:10.3389/fgene.2025.1599750)
Supplement: Supplementary file 1 [file DataSheet1.docx]

**Summary of the included studies**

| **Source** **of literature** | **Ethical issues** |
| --- | --- |
| Wasserman, 2004 | *Use of behavioural genetics by educational institutions:* lowering the intervention threshold – either broadly or selectively for low-MAOA children – would undermine parental autonomy, disproportionately target poor and working-class families, and infringe on children’s privacy by mandating non-medical genetic screening. A selective approach would create a moral paradox by prioritizing intervention for children deemed a future risk to society while neglecting equally maltreated high-MAOA children. More alarmingly, proposals for selective embryo implantation or abortion based on MAOA alleles raise eugenic concerns, threatening reproductive autonomy and reinforcing genetic determinism. Such policies risk shifting child protection from a rights-based approach to a utilitarian model of social control, where genetic predispositions dictate state intervention and reproductive choices |
| DeCamp and Sugarman, 2004 | *Risk of medicalization and geneticization:* concern that defining some behaviors as illnesses may lead to far-reaching effects, including prenatal diagnosis and pregnancy termination, altered perceptions of people with disabilities, and increased stigmatization of “abnormal” behaviors |
| Rothstein, 2005 | *Use of behavioural genetics by educational institutions*: first, school officials typically lack the expertise to accurately evaluate behavioral genetic technologies or determine when and how to incorporate genetic information into educational decisions. Additionally, schools often lack adequate privacy protections, increasing the risk of wrongful disclosure of sensitive genetic data. Second, there is a risk that behavioral genetic information will be overemphasized in assessing complex traits like cognition, potentially overshadowing environmental, social, and educational factors that play a crucial role in student development  *Employment*: similar to its application in education, the misuse of behavioral genetics in employment raises significant ethical concerns. If genetic information is improperly applied, it could lead to unjust exclusion of individuals from job opportunities based on perceived genetic predispositions rather than actual abilities or qualifications. This practice risks reinforcing discrimination, undermining merit-based hiring, and violating privacy and workers’ rights  *Insurance:* Behavioral genetic information could influence eligibility and pricing across various insurance sectors. In private health insurance, it might be used to predict an individual’s likelihood of requiring mental health services for psychiatric conditions or addictions. Disability insurance providers could assess genetic predispositions to behavioral health issues that increase the risk of temporary or permanent disability. Even in life insurance, genetic markers for risk-taking, impulsivity, or depression might be considered indicators of premature mortality, though some countries, like the UK, have implemented restrictions on the use of genetic data in underwriting. Additionally, behavioral genetic traits could be used to assess risk levels in automobile, household, or property insurance.  The use of such genetic information raises two key questions: scientific validity – how reliable and accurate are the actuarial predictions based on behavioral genetic research? Social and ethical implications – even if accurate, should private insurance companies be allowed to use genetic predispositions to determine coverage and rates, potentially leading to discrimination and reduced access to essential services?  Recommendations for responsible use: prevent misuse and overinterpretation – researchers should exercise caution in public statements and avoid overstating preliminary findings; improve public and media literacy – greater awareness is needed regarding past abuses and scientific limitations of behavioral genetics; ethical policy development – commercial and social institutions must consult experts and ethicists before integrating behavioral genetics into decision-making, ensuring it does not lead to stigmatization or exclusion; ensure confidentiality – given the sensitive nature of behavioral genetic data, strict privacy protections must be enforced to prevent misuse and discrimination |
| Popma and Raine, 2006 | *Interpretation of free will*: the integration of biological factors (e.g., prefrontal damage) into the understanding of antisocial behavior challenges traditional notions of free will and legal accountability. If such deficits impair impulse control, should an individual’s criminal responsibility be reconsidered? This perspective suggests that free will exists on a continuum, where greater biological impairment may correlate with diminished culpability.  This raises two opposing ethical concerns: from a conservative standpoint, reliance on biological explanations may lead to reduced sentences or exoneration of violent offenders, undermining justice and public safety; from a liberal perspective, biological profiling could lead to preemptive incarceration or discrimination against individuals deemed genetically predisposed to violence, even in the absence of criminal behavior |
| Savulescu et al.,2006 | *Compromising autonomy*: screening for behavioural traits risks compromising a child’s autonomy. It is sometimes argued that by making knowledge about a person’s genetic characteristics available to them, we restrict the range of choices that appear rational to the individual, thereby undermining the child’s “right to an open future”  *Discrimination:* if some particular selection choice became a dominant choice for the eradication of some particular trait, then those who are born carrying the unwanted trait would be discriminated against or devalued |
| Levitt and Manson, 2007 | *Coercion and stigmatization:* a significant ethical concern is that interventions targeting individuals or families may lead to coercion and stigmatization. Even if Quantitative Trait Loci (QTLs) associated with behavioral traits were evenly distributed across different social classes and ethnic groups, any genetic testing program would likely begin with families deemed “at risk” based on social and environmental criteria. This raises questions about fairness, discrimination, and the potential reinforcement of social biases under the guise of genetic risk assessment  *Responsibility:* Can evidence from behavioral genetics justifiably influence the attribution, withdrawal, or revision of moral and legal responsibility? For instance, a habitual or “career” criminal may have a genetic predisposition to behaviors that violate the law. However, the mere existence of such a predisposition does not, in itself, constitute a sufficient basis for assigning or absolving responsibility. This raises fundamental ethical and legal questions about the extent to which genetic influences should factor into assessments of culpability and the risk of undermining the principle of individual accountability |
| Berryessa et al., 2013 | *Determinism:* Fear that genetic explanations justify inequalities or limit social mobility. Risk of self-fulfilling prophecies if antisocial behavior is seen as inherited.  *Mental Illness and Stigma:* Debate over whether antisocial behavior should be classified as a mental illness. Risk of minimizing personal responsibility if labeled as a disorder. Stigma may negatively impact individuals diagnosed with antisocial tendencies.  Media often incorrectly associates mass violence with mental illness.  *Medicalization*: Labeling antisocial behavior as a medical condition may reduce stigma but also lessen personal accountability. Raises ethical concerns about using medication to control norm-defiant behavior. Potential risks of forced treatment, similar to past controversies on forced medication and chemical castration.  *Responsibility and Punishment:* Criminal responsibility relies on proving intent and mental state. Concerns that genetic evidence could undermine criminal liability. Genetic evidence may sometimes lead to reduced sentences but also to increased perceptions of dangerousness.  *Privacy and Discrimination:* Fear of genetic discrimination in employment, healthcare, and legal proceedings. DNA databases raise ethical concerns about consent and misuse of genetic information. Risk of profiling based on genetic predispositions rather than actual behavior.  *Risk and Dangerousness:* Courts may overestimate the predictive value of genetic markers for violent behavior. Concerns about labeling children as future criminals based on genetic screening.  *Surveillance and Regulation:* Growing concerns about the use of genetic data for preemptive crime prevention. Fear that genetic predispositions could be used for behavioral monitoring and regulation.  *Expertise:* Judges and juries often lack the scientific knowledge to assess genetic evidence. Risk of over-reliance on expert testimony without understanding its scientific limitations. Need for better education on genetic research in forensic settings. |
| O'Mahony and de Paor, 2017 | *Risk of misuse in criminal justice:* DNA samples stored in databases may be used not only for crime detection but also to assess the dangerousness and risk of convicted individual  *Human rights and stigmatization:* the implications of genetic testing for defendants remain largely unexplored, with concerns about discrimination and increased stigmatization, especially for those presumed to have a predisposition to criminal behavior.  *Genetic determinism*: the use of genetic science may lead to a shift towards genetic determinism, with individuals being labeled as having a “violence gene” or “criminal gene” despite the weak scientific basis for such classifications.  *Resurgence of eugenic thinking:* the increasing use of genetic information to isolate and target specific traits raises concerns about a revival of eugenic-style policies and attitudes  *Genetic discrimination:* the use of genetic data in legal and social contexts may lead to prejudicial treatment based on genetic predispositions  *Privacy violations*: unauthorized access, disclosure, and misuse of genetic information pose significant risks to the privacy and well-being of defendants and their families, potentially leading to physical, and psychological harm |
| Specker et al., 2017 | *Decreased tolerance for deviance*: expanding intervention opportunities may reduce societal and forensic tolerance toward deviant traits and acting out  *Mandatory interventions and consent*: some question whether interventions might become mandatory (e.g., as a condition for rehabilitation). Many emphasize the importance of voluntariness and informed consent, though forensic treatments often involve coercion. Some believe that biomedical interventions could offer solutions for treatment-resistant individuals but should be considered a last resort  *Passivity in treatment*: concerns arise that biomedical interventions could make the patient overly passive. Some argue that this passivity is ethically problematic because it neglects the underlying causes of aggressive behavior and fails to enhance self-awareness and self-management. Others worry that passivity could reduce long-term treatment effectiveness, as benefits may last only while the intervention is applied  *Crime prevention vs. Child well-being*: these programs may prioritize crime prevention over helping children or alleviating suffering, fostering paternalistic and coercive approaches that label some children as “risky”  *Reductionism and socio-political context*: ethical concerns include reductionism in explaining violent behavior, difficulties in interpreting neuroimaging in court, and risks of early detection programs, such as labeling, stigmatization, social control, and an excessive focus on risk  *Coercion and mental liberty:* the legitimacy of coerced biomedical interventions is debated. Some argue that forced incarceration already limits mental liberty, but others contend that neurointerventions more directly infringe on a person’s ability to reflect on and reject changes to their self-identity. While incarceration restricts movement, biomedical interventions may override cognitive autonomy in more profound ways |
| Ferioli and Picozzi, 2018 M. | *Psychological and social impact*: Predictive genetic tests can have significant psychological and social effects, such as anxiety, depression, or lifestyle changes. Counseling must address these implications to support informed decision-making  *Communication challenges*: Clear communication is crucial in explaining test results and their interpretation. Strategies must be developed to effectively convey complex genetic information to users  *Self-determination and rights*: Individuals have the right to decide whether to undergo testing, access their data, and control the dissemination of their genetic information. They also have the “right not to know” their genetic predispositions  *Responsibilities and duties*: Those who choose testing must consider potential personal and familial impacts, such as emotional distress, lifestyle changes, and procreative risks. Counseling should promote self-awareness and responsible decision-making  *Self-perception and social stigma*: Being labeled as “at risk” can affect an individual’s self-esteem and sense of identity. Societal expectations of physical and mental health can exacerbate feelings of being different or socially unacceptable  *Discrimination concerns*: Genetic information is sensitive and requires strict confidentiality. However, fears of discrimination based on genetic profiles persist, particularly in workplaces, insurance, and education. Some countries have laws prohibiting genetic discrimination, but enforcement and interpretation vary  *Insurance and employment*: In liberal economies, genetic testing has been used for workplace selection, career progression, and insurance coverage. While some European countries ban the use of genetic data in insurance, others have moratoriums or limited regulations, creating inconsistencies in protection  *Social stigma and exclusion*: Beyond formal discrimination, subtle forms of stigma and social exclusion based on genetic information are prevalent. The “DNA mystique” can lead to societal classification and control, fostering exclusionary practices in various social contexts  *No-stigma principle*: Emerging alongside non-discrimination, the no-stigma principle aims to address psychological hostility or discomfort toward those perceived as genetically “different.” This principle seeks to combat social exclusion and promote inclusivity.  *Personal freedom:* within the criminal justice system, the shift toward a preventive model could legitimize the restriction of personal freedom based solely on genetic predispositions to violence and crime, regardless of actual unlawful conduct. This approach could lead to selective policies of Lombrosian memory. |
| Glenn and McCauley, 2019 | *Ethical risks of individualized interventions*: while tailoring intervention programs based on biological information may enhance effectiveness, it raises ethical concerns related to stigma, discrimination, privacy, and equitable access to services  *Fairness and equality concerns*: differentiating service levels based on biological factors may violate fundamental principles of fairness and equality  *Negative impact of labeling*: Labeling a child as a “nonresponder” could alter the way parents and teachers interact with them, reinforcing the label. Clear communication is essential to emphasize that interventions are meant to support, not define, the child, and that biological factors are not fixed but modifiable  *Risk of miscategorization:* predictions will never be entirely error-free, posing the risk of misclassification. However, ongoing research can enhance predictive accuracy, and even a simple model incorporating a few factors could improve intervention outcomes beyond current effectiveness levels |
| Meurer, 2021 | *Risk of worsening health inequalities*: without proper regulation, epigenetics may exacerbate public health disparities rather than mitigate them. Cultural biases could shape funding priorities, favoring visible forms of violence while neglecting areas of greater need, reinforcing existing power structures.  *Stigmatization of disadvantaged populations*: identifying epigenetic risk factors more prevalent in marginalized communities may reinforce negative stereotypes, further marginalizing these groups and worsening social and health disparities.  *Justice and equity in intervention responsibility:* epigenetic findings raise complex ethical questions about who is responsible for acting on acquired information, particularly regarding screening, treatment, and resource allocation. Current tests can only suggest susceptibility to maladaptive behaviors in specific contexts, highlighting ethical concerns in their application  *Ethical concerns in testing and access* to care: mandating tests that could later subject individuals to interventions without ensuring treatment access raises serious ethical issues. Similar concerns apply when genetic links are disclosed without providing care, especially when treatment costs are high, leading to debates on resource allocation and state responsibility  *Need for transdisciplinary oversight:* while epigenetic insights support a larger role for public health, other sectors such as justice and medicine may also assert authority. This highlights the necessity of cross-sectoral coordination, as decisions on regulatory oversight are often shaped by political frameworks |
